# Inflammatory Responses Associated with the Induction of Cerebral Malaria: Lessons from Experimental Murine Models

**DOI:** 10.1371/journal.ppat.1003045

**Published:** 2012-12-27

**Authors:** Diana S. Hansen

**Affiliations:** The Walter and Eliza Hall Institute of Medical Research, Parkville, Victoria, Australia; University of Wisconsin Medical School, United States of America

## Introduction

Malaria is one the most serious infectious diseases of humans, with ∼500 million clinical cases annually. Most cases of severe disease are caused by *Plasmodium falciparum*, which is endemic in sub-Saharan Africa and throughout the tropics. Severe malaria is associated with a range of disease syndromes including respiratory distress, metabolic acidosis, hypoglycaemia, renal failure, and cerebral malaria (CM) [1]. The blood stage of the parasite is entirely responsible for malaria-associated pathology. Mature forms of *P. falciparum* blood-stage express parasitic proteins (such as *P. falciparum* erythrocyte membrane protein 1) on the surface of the infected erythrocyte, which allow them to bind to vascular endothelial cells and avoid clearance in the spleen. This process called sequestration induces obstructions in the blood flow and is postulated to result in hypoxia and haemorrhages that appear to be associated with the development of organ-specific syndromes such CM and placental malaria [1]. Many disease-association studies indicate that in addition to parasite sequestration, inflammatory responses also contribute to severe disease [1]. High TNF, IFN-γ, and IL-1β levels have been associated with disease severity [1]. The role of inflammatory cytokines in severe malaria is not completely understood, but they are thought to contribute to disease by up-regulating the expression of adhesion molecules such as ICAM-1 involved in the binding of parasitised red blood cells (pRBCs) to the vascular endothelium. Although parasite sequestration is the most common feature of patients succumbing to CM, postmortem examination has also revealed intra- and peri-vascular pathology including the presence of leukocytes within brain blood vessels [2]. These findings suggested that sequestration of host leukocytes might also contribute to the pathogenesis of some CM cases. Interestingly, recent findings revealed that increased levels of several inflammatory chemokines including MIP-1α and MIP-1β [3] and CXCL10 or IP-10 are associated with increased risk of severe malaria [4], suggesting a role for leukocyte trafficking in the etiology of human disease.

**Figure 1:**
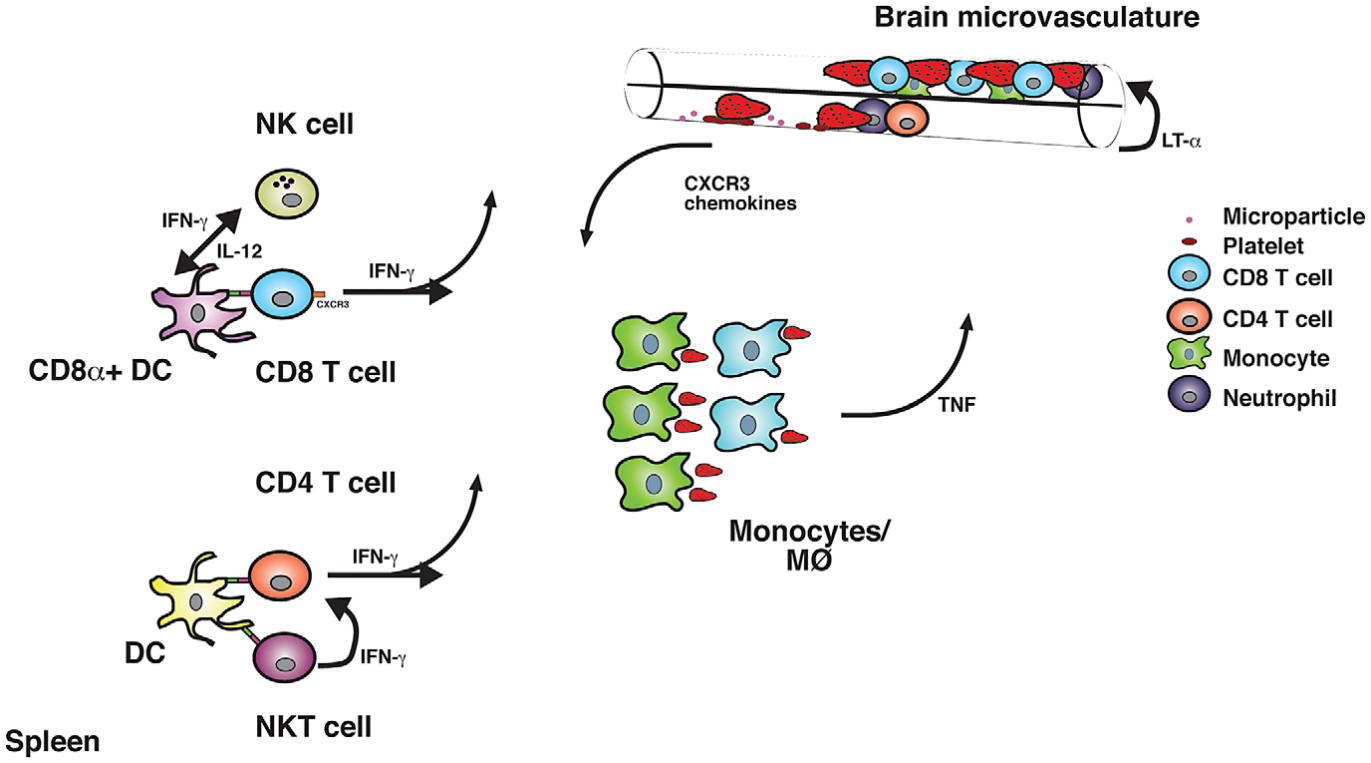
Inflammatory responses involved in the induction of ECM. After phagocytosis of pRBC, conventional DCs in the spleen present malarial antigens to CD4^+^ and CD8^+^ T cells. CD8α^+^ DC is the main subset involved in the cross-presentation of parasite-expressed antigens to CD8^+^ T cells. NK cells enhance the capacity of CD8α^+^ DCs to prime naïve CD8^+^ T cells. In turn, DC-derived IL-12 is required for efficient NK cell responses to infection. IFN-γ secretion by CD1-restricted NKT cells appears to favour Th1 polarization. High IFN-γ levels contribute to the activation of macrophages/monocytes, which phagocytose pRBC and secrete other inflammatory cytokines such as TNF. Systemic pro-inflammatory cytokine responses and pRBC in brain blood vessels activate the vascular endothelium. This results in the release of MPs, which might enhance pRBC accumulation within brain blood vessels. Platelets might also contribute to this process. Activated endothelial cells release cytokines and chemotactic factors, which facilitate the recruitment of inflammatory cells. Neutrophils migrate to the brain and have been shown to contribute to disease induction. Activated CD8^+^ T cells and to a lesser extent CD4^+^ T cells up-regulate the expression of chemokine receptors such as CXCR3, which allows them to migrate to the inflamed organ. Sequestered pRBC and leukocytes impair cerebral blood flow, which might result in hypoxia. Cytotoxic molecules released by inflammatory leukocytes compromise the integrity of the blood brain barrier, which results in oedema and haemorrhages associated with the onset of severe disease.

Clearly, the analysis of intravascular inflammation predisposing to CM in humans is limited to the examination of post-mortem samples. Thus, murine malaria models constitute a valuable tool to obtain detailed mechanistic information, which cannot be deduced from human studies. Much useful evidence on the inflammatory processes (Figure 1) contributing to the induction of CM has been provided by the *Plasmodium berghei* ANKA model. This rodent infection has several features in common with human disease and is the best available model of severe malaria. Like in humans, pRBCs have been found to accumulate in brains of susceptible mice during infection. Numerous leukocytes are also present in brain blood vessels of these animals. This article summarizes the main lessons learnt from murine malaria studies and illustrates how emerging information on inflammatory pathways predisposing to disease might open new avenues for the development of therapeutic strategies to alleviate severe malaria.

## Systemic and Organ-Specific Inflammation Mediated by Leukocytes

Inflammatory responses mediated by cytokines such as TNF [5], IFN-γ [6], LT-α [7], and effector cells including CD4^+^T [8], CD8^+^T [9], [10], NKT [11], and NK cells [12] have been shown to contribute to the development of experimental CM (ECM). CBA and C57BL/6 mice, predisposed towards Type-1 responses, are susceptible to the ECM, whereas BALB/c mice are resistant. C57BL/6 and BALB/c mouse strains differ in the expression of molecules encoded by a genetic region called the Natural Killer Complex (NKC), and it has been shown that the differential expression of these receptors in CD1d-restricted NKT cells influences their immunological behaviour in response to malaria and accounts for the degree of susceptibility to ECM [11]. The expression of C57BL/6 NKC alleles, which is associated with disease severity, appears to favour enhanced IFN-γ responses to infection [11].

The role of T cells in the pathogenesis of ECM has been extensively investigated. Antibody depletion studies as well as infection of β_2_-microglobulin^−/−^ mice demonstrated that CD8^+^ T cells contribute to the induction of ECM [8]. Cytotoxic CD8^+^ T cells have been found sequestered within brain blood vessels of *P. berghei* ANKA-infected mice [9], [10], and they appear to mediate CM via a perforin-dependent mechanism. The vast majority of inflammatory CD8^+^ T cells as well as CD4^+^ T cells and NK cells migrate to the brain of infected animals via a CXCR3-IP-10-dependent mechanism [13]–[15]. Brain-sequestered CD8^+^ T cells have been found to be specific for parasite-expressed model antigens [16]. Dendritic cells (DCs) are essential for the priming of T cell responses involved in the development of ECM [17]. Amongst these cells, CD8α^+^ conventional DCs are the main subset involved in the cross-presentation of parasite-expressed antigens to naïve CD8^+^ T cells [16].

CD4^+^ T cells have also been found in brain blood vessels of CM-affected mice [10]. They are not as abundant as CD8^+^ T cells, and it is not clear if they are parasite-specific or not. Nevertheless, in vivo depletion of CD4^+^ T cells with antibodies or genetic deletion of MHC class II molecules [8] also results in protection of susceptible mice from ECM. Recent work demonstrated that IFN-γ production by CD4^+^ T cells is required for the recruitment of CD8^+^ T cells to the brain of infected animals [18].

NK cells are important mediators of IFN-γ responses to malaria in humans and mice. Specific depletion of NK cells protects mice from *P. berghei* ANKA-mediated severe disease [12]. NK cells were shown to stimulate the recruitment of CXCR3^+^ T cells to the brain of malaria-infected mice in an IFN-γ-mediated manner [12]. More specifically, NK cells appear to stimulate the DC-mediated priming of naïve CD8^+^ T cells in response to *P. berghei* ANKA [19].

## Cytokine-Dependent Activation of the Brain Vascular Endothelium

IFN-γ as well as members of the TNF superfamily such as LT-α and TNF [20] are produced in large amounts during infection and play important roles in the activation of the vascular endothelium. ICAM-1, VCAM-1, and P-selectin become highly up-regulated on brain vascular endothelial cells during the development of ECM [1]. Moreover, ICAM-1- [21] and P-selectin- [22] deficient mice were found to be resistant to ECM. Leukocyte adhesion to the brain microvasculature was not inhibited in these animals. In contrast, the binding of platelets, which have been suggested to play a role in the development of CM in humans and mice, was substantially reduced in ICAM-1^−/−^ and P-selectin^−/−^ mice [23]. Whether genetic deletion of these adhesion molecules inhibits pRBC accumulation in the brain has not been investigated yet.

Cytokine-mediated activation of the vascular endothelium might also result in the release on microparticles (MPs). These submicron vesicles have been implicated in various neuroinflammatory conditions [24]. MPs were found in high numbers in patients with acute CM, they appear to increase the binding of pRBC to endothelial cells, and inhibition of their production in *P. berghei* ANKA-infected mice protects from the onset of the cerebral disease [24].

## The Contribution of Inflammatory Responses to Parasite Tissue-Sequestration

Accumulation of pRBC within brain blood vessels has been detected in CM-affected mice [25]. Unlike *P. falciparum*, in which the ligands responsible for cytoadherance have been extensively investigated, the mechanism by which pRBC accumulate in the brain during rodent malaria is still unknown. *P. berghei* ANKA transgenic parasites expressing luciferase have been generated [26] and are a valuable resource to address this question. Bioluminescence in mice receiving a synchronous infection for 22–30 h has been detected in lungs, spleen, and adipose tissue but not brain [26]. Although these findings initially implied that brain sequestration was not required for cerebral pathology in this model, the results could not exclude the possibility that the short infection period used in the study was insufficient for appropriate induction of inflammatory responses and activation of the brain vasculature, which appear to be required for parasite brain accumulation. In support of that view, parasite sequestration could be readily detected after 6–7 days of infection with luciferase-expressing lines in brains of intracardially perfused mice [15]. Moreover, parasite biomass in the brain was found to be associated with increased risk of CM [15].

The contribution of inflammatory responses to pRBC accumulation in the brain has been recently investigated [27], [28]. Consistent with previous observations [15], parasite sequestration in various organs, including the brain, was found to be strongly associated with the onset of ECM. Furthermore, CD4^+^ T cells, CD8^+^ T cells, and the cytokines IFN-γ and LT-α appear to directly promote this process [27], [28].

## Why Is It Useful to Have a Good Model of Severe Malaria?

There is an urgent need to develop adjunct therapies to improve the outcome of CM patients during treatment with antimalarial drugs. Good preclinical animal models provide a fast and cost-effective resource for assessment of new interventions. Although some interventions such as anti-TNF therapy that prevented ECM have not been effective in humans [29], other treatments such as L-arginine or NO administration appear to be protective in mice and humans [30], [31]. An important consideration that should be taken into account in the design of adjunctive therapies for severe malaria is that many inflammatory responses that contribute to disease are also involved in the control of parasite densities. Novel approaches such as inhibition of endothelial activation [32] and anti-chemokine therapies [15] are emerging as potential safe therapeutic alternatives, as they alleviate organ-specific inflammation without inducing a generalised immunosuppression of the host.
